# The Dividing Line Between Wildlife Research and Management—Implications for Animal Welfare

**DOI:** 10.3389/fvets.2019.00013

**Published:** 2019-02-05

**Authors:** Johan Lindsjö, Katarina Cvek, Elin M. F. Spangenberg, Johan N. G. Olsson, Margareta Stéen

**Affiliations:** ^1^Swedish Centre for Animal Welfare, Swedish University of Agricultural Sciences, Uppsala, Sweden; ^2^Department of Animal Environment and Health, Swedish University of Agricultural Sciences, Uppsala, Sweden; ^3^Department of Clinical Sciences, Swedish University of Agricultural Sciences, Uppsala, Sweden; ^4^Jämtlands County Administrative Board, Östersund, Sweden; ^5^Department of Anatomy, Physiology and Biochemistry, Swedish University of Agricultural Sciences, Uppsala, Sweden

**Keywords:** wildlife, research, management, animal welfare, 3R, legislation, ethical assessment

## Abstract

Wild animals are used for research and management purposes in Sweden and throughout the world. Animals are often subjected to similar procedures and risks of compromised welfare from capture, anesthesia, handling, sampling, marking, and sometimes selective removal. The interpretation of the protection of animals used for scientific purposes in Sweden is based on the EU Directive 2010/63/EU. The purpose of animal use, irrespective if the animal is suffering or not, decides the classification as a research animal, according to Swedish legislation. In Sweden, like in several other European countries, the legislation differs between research and management. Whereas, animal research is generally well-defined and covered in the legislation, wildlife management is not. The protection of wild animals differs depending on the procedure they are subjected to, and how they are classified. In contrast to wildlife management activities, research projects have to implement the 3Rs and must undergo ethical reviews and official animal welfare controls. It is often difficult to define the dividing line between the two categories, e.g., when marking for identification purposes. This gray area creates uncertainty and problems beyond animal welfare, e.g., in Sweden, information that has been collected during management without ethical approval should not be published. The legislation therefore needs to be harmonized. To ensure consistent ethical and welfare assessments for wild animals at the hands of humans, and for the benefit of science and management, we suggest that both research and management procedures are assessed by one single Animal Ethics Committee with expertise in the 3Rs, animal welfare, wildlife population health and One Health. We emphasize the need for increased and improved official animal welfare control, facilitated by compatible legislation and a similar ethical authorization process for all wild animal procedures.

## Introduction

Since the world's first animal protection legislation was established in England in 1822, several countries have implemented protection of animals as a part of the national legal framework. In spite of having a far-reaching legal Swedish framework for the protection of animals, there are shortcomings regarding the protection of wild animals, a situation not unique for Sweden (1, 2). The Swedish Animal Welfare Act (1988:534) includes all animals kept in captivity, but does not include free-ranging wildlife. However, if wild animals (vertebrates and cephalopods) are used for research, they are classified as research animals and are covered by the Animal Welfare Act and Swedish regulations for research animals (SJVFS 2017:40, case no L150 [L150]). The Directive 2010/63/EU (EU Directive) on the protection of animals used for scientific purposes (3) has been implemented in the Swedish legislation. Importantly, Sweden maintained a definition of research animals which also includes animals in scientific procedures where they are not necessarily exposed to any suffering. Gaining knowledge is fundamental in the Swedish legislation. It is the purpose, i.e., to obtain knowledge, that decides if an animal is a research animal. According to the EU Directive, it is not permitted to use wildlife in animal experiments, but competent authorities may grant exemptions if the purpose cannot be achieved by using animals bred for the purpose of research (Article 9.1, 9.2). Capture and handling must be carried out by competent persons and using methods which “do not cause the animals avoidable pain, suffering, distress or lasting harm” (Article 9.3). Staff who perform research procedures and handle the animals must be adequately trained (3). Research activities have to meet several requirements, including an authorization issued by the competent authority (in Sweden; Swedish Board of Agriculture) that allows the researcher to perform studies on animals, and an ethical approval for each research project from an animal ethics committee (AEC). One exception from ethical approval in the Swedish legislation (L150) includes scientific observations of wild animals which do not cause stress or suffering. Conversely, when wild animals are subjected to management activities (here defined as activities promoting the balance between the needs of wildlife with the needs of people through population, environmental and disease monitoring and control) no such authorizations are required, even though wildlife management often includes similar animal practices as research. Hunting in general is an integral part of managing wildlife in Sweden but is not defined as wildlife management within the scope of this article. The Swedish hunting legislation (Hunting Act [SFS 1987:259]) includes some welfare aspects on wildlife, except for animals used in research. The legislation states that wildlife shall not be exposed to unnecessary suffering during hunting, but does not express animal welfare or ethical requirements explicitly for management activities. In fact, neither the Swedish Hunting legislation nor the EU Directive *per se* mention or define the term “wildlife management.” This means that the welfare of wild animals used for research purposes is covered by the legislation, but not the welfare of wild animals subjected to management activities.

Irrespective of the intention—research or management—the welfare of wild animals subjected to capture, anesthesia, handling, sampling, marking and sometimes selective removal (i.e., culling) may be compromised (2, 4). Negative impact on individual animal welfare can affect research quality as well as management results at group and population levels (5, 6). It is often difficult for responsible authorities to define the dividing line between wildlife research and management, and to identify the correct legislation for different situations. Moreover, contrary to research activities, it is difficult to control wildlife management activities from an animal welfare perspective. As a result, some wild animals are more protected than others, depending on which category they belong to. The aim of this review is to discuss the differences, similarities and overlap between wildlife research and management and its effects on animal welfare, with Sweden as an example.

## The Gray Area Between Using Wild Animals for Research or for Management Purposes

The purpose of research is to answer a scientific question. When an activity is performed purely from a management perspective, for example preserving an animal species or monitoring population health, it is not necessarily classified as an animal experiment. The EU Directive does not apply to “practices undertaken for the primary purpose of identification of an animal” nor to “practices not likely to cause pain, suffering, distress or lasting harm equivalent to, or higher than, that caused by the introduction of a needle in accordance with good veterinary practice” (Article 1.5) (3). Whether or not a procedure falls under the EU Directive is based on the purpose of the procedure and if the procedure causes negative welfare effects above the threshold (3, 7). Red foxes (*Vulpes vulpes*) are selectively removed for the protection of arctic foxes (*Vulpes lagopus*) in the alpine terrain of Sweden. If data collected during this management process is used to gain scientific knowledge, the procedure should be considered animal research. If so, it should be subjected to an ethical review and project approval by the competent authority. Data from management activities, e.g., assessing population size, migration behavior, home ranges and health, are often published by governmental authorities. In Sweden, publishing data from a procedure, irrespective of its intention, should be considered research. Additional information may be collected as part of a management procedure, including clinical and physiological variables to ensure health and welfare on anesthetized animals (8, 9). If these data are analyzed and published, it is considered as research in several European countries (7). It is disadvantageous for science to be unable to use collected data because of lack of ethical assessment and project approval. The opposite situation can also occur when authorities in Sweden want to use data from ongoing research (e.g., GPS positions) for management purpose, like tracking down wolves (*Canis lupus*) for culling (10). This will not be permitted if culling is not clearly stated as a purpose in the ethical approval (11). Discussions about the gray area are also held in Norway regarding marking of wild reindeer (*Rangifer tarandus tarandus*) for identification purposes (7). The challenge of an unclear dividing line between research and management in relation to the EU legislation was recognized during an international consensus meeting on the use of wild animals in field research (NORECOPA, 2017) (7). The EU Directive emphasizes the 3Rs—Replacement, Reduction, and Refinement (3, 12). A procedure such as marking an animal for identification and tracking involves capture, anesthesia and the placement of a tracking device on the animal, e.g., a collar or tag, or a transmitter in the abdomen (13–15). Even if the primary cause is identification, it is very likely that such a procedure may cause effects that are at least as negative to animal welfare as the insertion of a needle, i.e., stress, fear, and pain. In fact, such a procedure may not be defined as the least invasive method for identification (4), and would be scrutinized from a 3R-perspective if classified as a regulated procedure requiring an ethical assessment and permission from the competent authority. Ringing of birds is an important tool for population monitoring. The ringing procedure does not need ethical approval in Sweden if the procedure only includes capture, taking measures and applying a leg ring (16). There are some risks associated with bird ringing (17) and it can be argued that the stress of mist net capture and handling probably has a greater negative impact than the pain of a needle for many birds. In comparison, ethical permission is needed for all survey test fishing, i.e., using electric fishing and nets. The Swedish legislation is not consistent, i.e., catching fish for population assessments needs approval by the competent authority and AEC but capturing birds does not. Hence, there are gray areas regarding which actions are defined as animal research or wildlife management.

### The Importance of Ethical Assessment, Animal Welfare and the 3Rs in Wildlife Research and Management

Research projects that fall under the scope of the EU Directive must pass an ethical evaluation for approval (3). The project evaluation must include a harm-benefit analysis with regard to animal suffering and the predicted gain for society. In Sweden, there are six regional AECs. Each committee consists of 14 members. The chairman and vice-chairman are lawyers and the rest are equal numbers of researchers (or experimental animal technicians) and laymen. The Animal Ethics Committees primarily assess the use of traditional laboratory animals in biomedical research (18, 19). It is a recurring problem that the legislation is less adapted to research on wild animals. One example is the approved euthanasia methods which the AECs have to grant exemptions from when researchers catch fish in nets. The fish die slowly in the nets, which is not an accepted euthanasia method for research animals but is a standard method for population assessments of fish. Another example is using the measure of pain and stress equal to the insertion of a needle as the cut-off point for invasive procedures. In wildlife studies, pain and suffering are often not comparable to biomedical studies in terms of the procedures used. More importantly, wild animals may fare at least as badly from capture and handling since they have neither training nor any relationship with humans. According to the EU Directive, research projects must be planned according to the principle of the 3Rs (3, 12) which means that if there are no available alternatives to using animals, the number of animals should be the least possible to achieve statistically significant scientific results and that procedures should be performed in the most humane way possible. The 3Rs were originally designed for laboratory animals kept in research facilities (12), but are also applicable to free-ranging wildlife (20, 21). Species, research purposes and design, environment, and possibilities for close long-term monitoring of animals differ from those in traditional laboratory settings (5, 22). Nevertheless, replacement with computer simulations and environmental-DNA, reduction through optimized experimental design and sharing of data, and refinement with better methods of capture, anesthesia, handling, marking and design of equipment such as transmitters are examples of 3R strategies in wildlife research (20, 23, 24). Scrutiny of capture methods and how to define humane end-points for research on wild animals must be considered by the AECs. A humane end-point can, for example, be the maximum time allowed for helicopter chase of an animal or the number of attempts to descend upon the animal before immobilization. The project plan should include a description on how animals should be treated if they are injured when captured, and a plan for euthanasia if an animal cannot successfully be treated. The project needs to monitor the animals once released whenever possible in order to ensure not only their immediate survival but also their viability (e.g., that social animals reunite with their group) (23). In fact, the 3Rs should be systematically applied throughout the wildlife research project, from planning of the project to publishing of data (23, 25).

For management purposes, the application of the 3Rs and evaluation of suffering and other welfare criteria within ethical assessments are not legally required. While the 3Rs are increasingly recognized in wildlife research (21), they are also applicable in wildlife management (6, 26). Crozier and Schulte-Hostedde (26) discussed animal welfare and ethical implications of wildlife disease management. The authors suggested indirect management practices on wildlife populations (e.g., fences to minimize contact, habitat management) rather than culling to prevent disease transmission between wildlife and domestic animals, and using the most humane culling methods on a minimal number of animals. Merbourg et al. (27) compared attitudes toward and methods used in rodent pest control and animal research. They proposed using methods to repel rodents from entering a specific area and using the most animal welfare friendly control methods.

Members of the AECs (or IACUCs [Institutional Animal Care and Use Committees] in America) are often not familiar with the specific issues of wildlife research (5, 22). This lack of wildlife expertise in the AECs is a problem that occurs in several countries. Sikes and Bryan (28) describe the situation in America and the unique issues when using wild animals for research. They state that the IACUCs should have special tools and competences to be able to fulfill the task of wildlife project review. The lack of expertise can unfortunately result in failing to ask the important questions to the investigators. Examples of such questions include asking how a transmitter is aerodynamically designed, rather than just asking how much the transmitter weighs in relation to the weight of the animal, or how the transmitter can affect movement and health of the animals (15, 29), or the short- and long-term risks on health and welfare from capture and handling (4). The research procedures in traditional laboratory settings affect animals confined in a controlled environment. In contrast to laboratory settings, it is not always feasible to monitor animals released back to the wild (20). Importantly, this may also have implications on a larger scale; short- and long-term effects of capture, handling and identification, relocation, selective removal, and unintentional disease transmission, may affect wildlife populations, environmental health and biodiversity, domestic animals and humans, i.e., One Health (5, 30, 31).

### Reduced Possibilities to Control Management Activities

The County Administrative Boards (CABs) are the competent authorities for carrying out official animal welfare controls in Sweden. In order to be able to perform these controls, the CABs need to be aware of what kind of animal activities, including research, are being carried out within the county. The gray area between research and management of wild animals complicates the official animal welfare control in Sweden. For the capture of wild animals, an authorization from the Swedish Environmental Protection Agency is required. Procedures carried out on animals (injections, blood sampling, anesthesia, surgery, etc.) can be permitted based on species preservation, i.e., management. If the procedure includes collection of data that can be used for a scientific purpose, it also requires permission from the Swedish Board of Agriculture in combination with an ethical approval from an AEC. The ethical approval is communicated to the CAB. If permission from an AEC to use wild animals in a research-like management situation is lacking, the CABs have no way of knowing that activities involving animals occurs. Such activities involving capturing, handling, sampling and marking of wild animals are not controlled by the CAB.

According to the Swedish Animal Welfare Act, animals in the care of humans must not be exposed to unnecessary suffering. Procedures that have been approved by an AEC are not considered to cause unnecessary suffering. However, animals subjected to invasive procedures (such as anesthesia) that have not been approved by an AEC, are considered to be suffering unnecessarily, unless the procedure has a veterinary justification for the individual animal. It can be argued that wild animals subjected to invasive management procedures when in temporary human care should fall under the Animal Welfare Act. In line with this reasoning, not only are the management activities unknown to the CAB, but they may also directly conflict with the Animal Welfare Act. The aforementioned example shows the difficulties when the legislation is unclear, and it opens up for different situation-based interpretations.

## Conclusions and Recommendations

Unclear and sometimes conflicting legal requirements and policies complicate the definition of a dividing line between wild animal research and management in Sweden, like in several other European countries (7). Hence it is difficult to determine into which category—research or management—an animal belongs, and if an ethical review of the animal procedure is needed. It is crucial that the competent authorities conduct a gap analysis between different legislations, e.g., in Sweden the legislations concerning animal welfare, hunting and fishing, and make them compatible. Wild animal management as such should be defined in the legislation and be subjected to animal welfare requirements similar to wildlife research.

The dividing line between research and management is hard to interpret. All procedures involving wildlife in research as well as management should undergo an ethical harm-benefit assessment for approval. The approval is not only beneficial from an animal welfare perspective, but will also facilitate the use of collected data, regardless of which category the handling of the wild animal has been defined as during the procedure. Within the current ethical project assessment and approval system, the knowledge of wild animal welfare and ethics is limited and needs to be improved (5). We therefore suggest that the assessment should be performed by one single AEC specialized in wildlife, with expertise in animal welfare, animal ethics, wildlife population health and One Health. This would ensure a similar ethical and welfare assessment for all wildlife. A completely new ethical committee could be created for this purpose. Alternatively, one of the existing AECs could specialize in wild animal practices by incorporating researchers with field experience, ethologists, biologists, lawyers, and public health experts.

We also suggest increased and improved official animal welfare controls of wildlife research and management procedures through harmonized legislation and facilitated by a mandatory authorization of animal procedures, based on ethical review.

Suggested changes and improvements would increase stakeholders' and public insight into, and understanding of, research and management procedures, and how these activities align with a harmonized legislation.

## Author Contributions

JL, KC, ES, JO, and MS all contributed equally to the problem description and discussion, and manuscript revision. All authors read and approved the submitted version.

### Conflict of Interest Statement

The authors declare that the research was conducted in the absence of any commercial or financial relationships that could be construed as a potential conflict of interest.
